# Epidemiology of multiple sclerosis in Central Europe, update from Hungary

**DOI:** 10.1002/brb3.1598

**Published:** 2020-03-20

**Authors:** Tamás Biernacki, Dániel Sandi, Zsanett Fricska‐Nagy, Zsigmond Tamás Kincses, Judit Füvesi, Rózsa Laczkó, Zsófia Kokas, Péter Klivényi, László Vécsei, Krisztina Bencsik

**Affiliations:** ^1^ Department of Neurology Faculty of General Medicine Albert Szent‐Györgyi Clinical Centre University of Szeged Szeged Hungary; ^2^ MTA ‐ SZTE Neuroscience Research Group Szeged Hungary

**Keywords:** 2017 McDonald criteria, epidemiology, multiple sclerosis, phenotypic classification, treatment status

## Abstract

**Objectives:**

Not so long ago, a novel phenotypic classification of multiple sclerosis (MS) and revisions to the McDonald diagnostic criteria were published. Good quality, standardized, and therefore comparable epidemiological data from the Central European region altogether are scarce, and data based on the aforementioned criteria are nonexistent; thus, an update is needed.

**Materials and Methods:**

Patients residing in Csongrád county with a definitive diagnosis of MS according to the 2017 McDonald criteria were included and evaluated by the 2014 revised phenotypic classification.

**Results:**

A total of 420 patients were included, of whom 313 were females (female/male ratio 2.925:1). Standardized prevalence was 101.8/100,000, and incidence was 4.44/100,000. Relapsing–remitting disease type was identified in 288 (68.57%) cases, of which 230 patients (79.86%) were treated and of which 202 patients (87.8%) showed no disease activity with their current treatment. Progressive disease type was seen in 132 (31.43%) cases, with 72 patients (54.54%) receiving treatment. More than half of the treated patients (178, 57%) were administered platform therapies, while 134 (43%) received highly active disease modifying therapies.

**Conclusion:**

The prevalence of MS in Hungary similarly to other countries shows a constant increase in the past decades. The majority of our patients received treatment and had a stable disease while being treated. The distribution of disease courses, phenotypes, and treatment status fell in line with data in the literature based on MS registries with a large number of participants. Ours is the first study to give epidemiological data based on the most recent McDonald criteria and phenotypic classification from the Central European region.

## Graphical abstract

Recently, a new phenotypic classification of multiple sclerosis (MS) and revisions to the McDonald diagnostic criteria and therapeutic guidelines were published. Good quality, standardized, and therefore comparable epidemiological data from the Central European region altogether are scarce, and data based on the aforementioned principles are nonexistent. Based on these, we examined prevalence and incidence of MS as well as the treatment, and disease activity status of the MS patients residing in Csongrád County, Hungary.

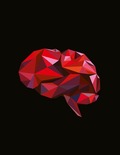

## 1 INTRODUCTION

Multiple sclerosis (MS), although considered as a rare disease, is the most common chronic, autoimmune, demyelinating, and neurodegenerative disease of the central nervous system and is the second most common cause (after traumatic injury) of permanent disability among young adults (Alastair Compston et al., 2005). In recent years, the therapeutic palette has expanded considerably; nowadays, there is at least one drug available for every disease course. Adjacent to the introduction of new disease‐modifying therapies, a novel standpoint is gaining ground regarding the therapeutic strategy of MS.

In 2017, the most recent revisions to the McDonald diagnostic criteria were published (Thompson et al., 2018). According to these, the diagnosis of clinically definitive multiple sclerosis can now be set even in patients who previously were classified as clinically isolated syndrome (CIS) patients, based on solely one clinical event supported by magnetic resonance imaging (MRI) and/or cerebrospinal fluid (CSF) examination. Furthermore evidence suggests that the future disease activity can be predicted from the baseline MRI parameters (Davda, Tallantyre, & Robertson, 2019).

In addition to the newly introduced diagnostic criteria, the very first comprehensive therapeutic guideline was also published in 2018 (Montalban et al., 2018a, 2018b), which breaks with the previous escalative therapeutic strategy of starting with less potent platform therapies (interferons, glatiramer acetate, dimethyl fumarate, teriflunomide) which, if turn out to be inefficient, can be switched onto more potent disease modifying treatments (DMT). On the contrary, the new guideline recommends the treatment of every patient as soon as possible, with disease modifying treatments matching the patient's disease activity, advocates the use of highly active DMTs (HAMDT—fingolimod, natalizumab, ocrelizumab, cladribine, alemtuzumab, and mitoxantrone) as a first choice for patients with highly active disease. Furthermore, a new phenotype classification was also established (Lublin, 2014). It maintained much of the basic attributes of the originally defined disease courses; however, in contrast to the previous classification system, it lays a much greater emphasis on the activity of the disease and the gradual worsening of symptoms in order to categorize the disease into different phenotypes. It recognizes two major courses of the disease, one being the relapsing–remitting (R‐R and CIS) type, the other being the progressive type (Lublin, 2014). Depending on the clinical and imaging presentation, a patient's disease diagnosed with CIS can be stable and show no activity, or can be clinically and/or radiologically active, in which case the disease is considered relapsing–remitting. Similarly, an R‐R disease can be stable or can show clinical and/or radiological activity. Likewise, patients with progressive diseases can be split into two groups whether activity (new, or unequivocally enlarging T2 lesions and/or contrast enhancement can be seen on MRI scans or a clinical relapse) is present (i.e., progressive disease with or without activity). These two groups can further be divided into categories based on the presence or the lack of continuous worsening of symptoms (i.e., progressive disease type with or without progression; Lublin, 2014).

Previously, when disease subtypes were classified based on clinical courses and on Expanded Disability Status Scale (EDSS) scores (Kurtzke, 1983), no clear cut margin was defined where the relapsing–remitting phase ended and secondary progressive (SP) phase (most commonly still with relapses) began. The EDSS score of R‐R patients traditionally ranged from 0 to 5.5, whereas it laid between 3.5 and 10.0 for SP patients. From previous large scale studies exploring the natural history of the disease (Scalfari et al., 2010), and epidemiological studies, it is known that no matter how long it took a patient from disease onset to reach the turning point between relapsing and progressive state; the disease advances roughly with the same speed in all patients thereafter (Leray et al., 2010; Scalfari et al., 2010). Irreversible axonal injury and transition into a progressive disease course occur approximately at an EDSS score of 4 (Lorscheider et al., 2016) and therapeutic window to stabilize a patient's illness and prevent transition is before this (Correale, Gaitan, Ysrraelit, & Fiol, 2017); with drugs currently at our disposal, we can significantly prolong the time between disease onset and irreversible axonal injury. The new phenotypic classification gives pivot to clinicians to assess the disease activity of their patients and to choose the most suitable DMT for them, also when to change, recognize if a given treatment has become inefficient. Recently published data shows that the biggest socioeconomical and financial burden on a society and healthcare giver is put on by the secondary progressive MS population, almost double that of the relapsing–remitting population (Purmonen, Hakkarainen, Tervomaa, & Ruutiainen, 2020). The majority, roughly 2/3rd of the total expense, fell into the categories of direct nonmedical costs and productivity loss, driven mainly by the early retirement of patients with secondary progressive disease (Purmonen et al., 2020). Up to date, comparable epidemiological data are needed according to the novel phenotypic classification system in order to assess the therapeutic, medical, and financial needs of patients with MS on a population basis (Kingwell et al., 2013), since many of the patients previously categorized with an R‐R disease now fall into the secondary progressive disease category according to the new classification system and therefore require a fundamentally different therapeutic approach, than before.

The aim of our study was to provide a picture of disease development (comparable age‐, and sex‐specific crude, and standardized prevalence and incidence of MS) from a previously surveyed demographic region and also to provide information about the disability state (measured by the EDSS score) as well as treatment and disease activity status of our patients almost a decade after the introduction and eventual widespread use of HADMTs based on the novel phenotypic classification system proposed by Lublin et al (Lublin, 2014).

## 2 PATIENTS AND METHODS

### 2.1 Study area

All of our patients were residing in Csongrád County, and point prevalence was determined on the prevalence day of 1 January 2019. Incidence data reflect the incidence of diagnosed patients (not disease onset) in the given time period. Csongrád County is located in the southeastern region of Hungary in the temperate zone, with an area of 4,262.68 km^2^ and humid continental climate.

According to the latest census performed by the Hungarian Central Statistical Office on 1 January 2019; 399,012 (accounting for 4.08% of the total population) people lived in Csongrád County, of which 189,420 were males and 209,592 females (http://www.ksh.hu).

### 2.2 Data collection

All of the enrolled patient's data (demographic, radiologic, and clinical) were extracted from the MS register maintained and updated by the MS outpatient clinic of the Department of Neurology at the University of Szeged since 1996 (Bencsik et al., 2017; paper based from 1996 to 2013, electronic from 2013), every patient's data are immediately updated after each visit to the MS clinic. All data shown in the present article represent the state of the patients on the prevalence day, and disease duration was calculated from the onset of each patient's disease. The study was approved by the Ethics Committee of the Faculty of Medicine, University of Szeged (207/2015‐SZTE). All participants gave their written informed consent in accordance with the Declaration of Helsinki. For the statistical analyses, we used SPSS version 22.

### 2.3 Diagnosis and follow‐up examinations

The diagnosis was established by the given patient's neurologist. Until 2001, the criteria of Poser were applied (Poser et al., 1983), and in subsequent years, always the latest available criteria of McDonald were used. All patients with diagnoses of MS according to Poser (Poser et al., 1983), and previous McDonald criteria (McDonald et al., 2001; Polman et al., 2005, 2011), were reviewed according to the latest McDonald criteria (Thompson et al., 2018).

At the time of diagnosis, in addition to physical examination, an MRI scan of the brain and, if necessary, the spinal cord was conducted (using a 1.5 T MRI scanner for both the brain and spinal cord before 2017, and only for the spinal cord after 2017, when a 3 T MRI scanner was implemented for brain imaging, always in adherence to the latest MAGNIMS guidelines; Filippi et al., 2016). A lumbar puncture was made to acquire CSF to be analyzed in our clinic's accredited laboratory (according to ISO 9002 standards) by the means of laser nephelometry for the quantitative determination of proteins, isoelectric focusing, and IgG immunoblotting for the detection of oligoclonal bands (OGP). Also, in necessary, visual‐evoked potential, somatosensory‐evoked potential, and brainstem‐evoked response audiometry tests were conducted.

After the diagnosis was established, all CIS, R‐R patients (according to the classical phenotype classification), and all other patients still receiving any kind of disease modifying therapy were routinely examined every 3 months. Every other patient (with progressive disease types, and patients without a DMT) was re‐evaluated at least annually. In the event of a relapse, an unscheduled, out‐of‐turn appointment was always provided for the patients. All patients diagnosed with CIS are advised to attend a routine check‐up annually indefinitely, unless another disease responsible for their symptoms is diagnosed or disease activity presents, from which point the regular visit schedule is recommended (i.e., at least every 3 months). During every control check‐up, the patient's current neurological status, EDSS score, and the clinical form of the disease were laid down and revised in the patient records. After the baseline MRI examination, control MRI scans were conducted on a regular basis; always in adherence with the SMPC of a DMT, a given patient is receiving, but at least yearly. In the event of a relapse before the initiation of intravenous corticosteroids, a contrast enhanced MRI scan was always conducted. A control brain MRI scan was done to all the patients presenting with CIS disease type both 3 and 6 months after the appearance of the first symptoms, and yearly thereafter. Every patient's medical chart participating in our study has been reviewed by four neurology specialists independently and was excluded from it, if at least one specialist felt uncertain about the diagnosis.

According to the aforementioned principles and in line with data from recent large scale studies, all patients who previously fell into the relapsing–remitting disease type were re‐evaluated for secondary progressive disease course using the objective, 3‐strata criteria proposed by Lorscheider et al. for defining secondary progressive MS (Lorscheider et al., 2016).

## 3 RESULTS

Our database registered 420 patients with MS on the prevalence day, 107 males and 313 females (the female–male ratio was 2.92:1). In the 6 years since our last epidemiological data collection from 2013, 108 new MS cases were diagnosed. In that period, 30 patients had died and 28 had moved away from the geographical area. The crude prevalence of MS for the whole cohort was 105.3/100,000, 56.5/100,000 for men and 149.3/100,000 for women (Table 1). The standardized prevalence was 101.8/100,000. Age‐, and sex‐adjusted, standardized prevalence was 53.9/100,000 for men and 144.8/100,000 for women. The 2013 European standard population was used for the standardization (Table 1).

**Table 1:** Age‐, and sex‐specific and standardized prevalence of multiple sclerosis in Csongrád county on the 1 January 2019

Age‐group (years)	Men		Women		Total	
	Cases	Prevalence	Cases	Prevalence	Cases	Prevalence
0–14	0	0.0	0	0.0	0	0.0
15–19	0	0.0	1	10.3	1	5.0
20–24	1	8.0	3	24.2	4	16.0
25–29	4	28.5	19	142.9	23	84.1
30–34	9	71.7	29	250.4	38	157.4
35–39	16	121.7	30	234.6	46	177.3
40–44	12	70.4	47	285.4	59	176.0
45–49	20	138.7	37	257.9	57	198.2
50–54	12	92.8	37	277.5	49	186.6
55–59	7	63.6	36	290.6	43	183.8
60–64	11	83.7	34	211.2	45	153.9
65–69	13	117.8	19	126.8	32	123.0
70–74	0	0.0	14	111.2	14	67.0
75–79	0	0.0	4	39.2	4	24.7
80–84	1	30.0	2	28.4	3	28.9
85–	1	41.1	1	16.2	2	23.3
Crude^a^	107	56.5	313	149.3	420	105.3
Age–sex‐adjusted^b^		53.9		144.8		101.8

Note

The average incidence of MS for the examined period was 4.44/100,000, 2.44/100,000 for men, and 6.25/100,000 for women, respectively.

^a^ Crude prevalence per 100,000 persons.

^b^ Standardized prevalence per 100,000 persons (the 2013 European standard population was used as reference population in the direct standardization).

Pursuant to the “old” disease course classification, our cohort comprised of 12 CIS (2.86%), 276 R‐R (65.71%), 102 SP (24.29%), and 30 primary progressive (PP; 7.14%) patients. When stratified by gender, no difference was seen regarding the distribution according to disease course between the groups (*p* = .166, data not shown). The average age for the whole cohort was 48.83 years (±10.64 years), age at diagnosis was 34.15 years (±10.64 years), average disease duration was 14.57 years (±10.59 years), and average EDSS score was 2.8 points (±2.44; Table 2).

**Table 2:** Characteristics of the whole cohort and stratified by disease type and disease activity

		Disease duration			EDSS			Age at diagnosis			Age		
		Mean	Median	SD	Mean	Median	SD	Mean	Median	SD	Mean	Median	SD
Cohort (420, 100%)		14.57	12.0	10.59	2.804 (0–9.5)	2.00	2.44	34.15	34.00	10.64	48.83	48.00	13.23
R‐R disease (288, 68.57%)	R‐R + CIS (288)	11.73	10.0	8.372	1.375 (0–3.5)	1.50	1.08	32.58	32.00	9.933	44.42	44.00	11.55
	CIS (12; 4.16%)	11.25	7.5	8.21	0.083 (0–1.0)	0.00	0.29	30.67	25.00	12.94	42.50	39.50	13.51
	R‐R (276; 95.84%)	11.75	10.0	8.39	1.431 (0–3.5)	1.50	1.07	32.67	32.00	9.80	44.51	44.00	11.48
	A‐T (28; 9.72%)	9.39	8.0	6.30	1.821 (0–3.5)	2.00	0.85	33.07	34.50	9.25	42.46	44.00	9.46
	A‐NT (15; 5.2%)	8.53	7.0	9.30	1.5 (0–3.5)	1.00	1.13	35.80	33.00	8.59	43.33	46.00	11.39
	NA‐T (202; 70.13%)	11.55	10.0	7.52	1.394 (0–3.5)	1.50	1.08	31.81	31.00	9.87	42.53	43.00	10.79
	NA‐NT (43, 14.93%)	15.19	10.0	7.52	0.953 (0–3.5)	1.00	1.08	34.77	34.00	10.82	50.28	48.00	14.57
Progressive disease (132, 31.43%)	PP + SP (132)	20.77	21.0	12.20	5.92 (3.0–9.5)	6.00	1.46	37.58	37.00	11.33	58.44	60.00	11.48
	PP (30; 22.73%)	10.17	8.0	8.11	5.617 (3.0–9.5)	6.00	1.73	48.33	48.00	9.73	58.53	60.50	10.24
	SP (102; 77.27%)	23.88	24.0	11.44	6.01 (4.0–9.0)	6.00	1.37	34.42	34.00	9.74	58.41	60.00	11.86
	A‐NP (8; 6.06%)	14.25	14.5	10.08	5.313 (3.5–8.5)	5.00	1.65	35.13	37.00	13.23	49.38	47.50	8.99
	A‐P (23; 17.42%)	15.17	13.0	10.49	5.652 (3.0–7.5)	6.00	1.28	34.74	35.00	10.35	49.91	50.00	10.46
	NA‐P (48; 36.36%)	22.06	22.5	12.56	6.542 (3.0–6.5)	6.50	1.55	40.17	38.50	12.49	62.33	63.00	11.16
	NA‐NP (53; 40.15%)	23.00	24.0	12.04	5.566 (3.0–9.0)	5.50	1.25	36.85	36.00	10.11	59.98	62.00	10.05

Note

The disease characteristics of our whole cohort as well as stratified by disease phenotype and disease activity. Disease duration, age at diagnosis, and patient age are given in years.

Abbreviations: A‐NP, active–not progressive (patient showing disease without progression); A‐NT, active–not treated (patients showing disease activity without receiving treatment); A‐P, active–progressive (patient showing disease and progression at the same time); A‐T, active–treated (patients showing disease activity despite receiving treatment); CIS, clinically isolated syndrome; NA‐NP, not active–not progressive (patient not showing disease activity nor progression); NA‐NT, not active–not treated (patient not showing disease activity without receiving treatment); NA‐P, not active–progressive (patient not showing disease activity while showing progression); NA‐T, not active–treated (patient not showing disease activity while receiving treatment); PP, primary progressive disease; R‐R, relapsing–remitting; *SD*, standard deviation; SP, secondary progressive disease.

According to the novel phenotypic classification, 288 (68.57% of the whole population) of our patients had a relapsing–remitting disease type. Their average disease duration was 11.73 (±8.37) years, age was 44.42 (±11.55) years, age at diagnosis was 32.58 (±9.93) years, and average EDSS score was 1.38 (±1.08) points. Twelve people (4.16%) had only a single attack (CIS). When stratified by phenotype and treatment status, 28 person's disease (9.72%) had showed activity despite treatment (active–treated arm, A‐T), their average disease duration was 9.39 (±6.30) years, age 42.46 (±9.46) years, age at diagnosis 33.07 (±9.25) years and average EDSS score 1.82 (±0.85) points. The active–not treated arm (A‐NT) comprised of 15 people (5.2%), their average disease duration, age, and age at diagnosis were 8.53 (±9.30), 43.33 (±11.39), and 35.80 (±8.59) years respectively, and mean EDSS score was 1.50 (±1.13) points. Our study included 202 (70.13%) patients whose disease was inactive while being treated (not active–treated arm, NA‐T). Their mean EDSS score was 1.39 (±1.08) points, and average age, age at diagnosis, and disease duration were 42.53 (±10.79), 31.81 (±9.87), and 11.53 (±7.52) years, respectively. A total of 43 people (14.93%) had inactive disease without treatment (not active–not treated arm, NA‐NT), of whom 20 patients had an isolated optic neuritis, but with additional diagnostic measures according to the latest McDonald criteria the diagnosis of definite MS could be made. Their average disease duration, age, and age at diagnosis were 15.19 (±7.52), 50.28 (±14.57), and 34.77 (±10.82) years and a mean EDSS score was 0.95 (±1.08) points (Tables 2 and 3).

**Table 3:** Distribution of patients by disease type and disease activity across the EDSS scale

		EDSS																		
		0	1	1.5	2	2.5	3	3.5	4	4.5	5	5.5	6	6.5	7	7.5	8	8.5	9	9.5
R‐R disease (288, 68.57%)	CIS (12; 4.16%)	11	1	0	0	0	0	0	0	0	0	0	0	0	0	0	0	0	0	0
	R‐R (276; 95.84%)	71	52	35	58	22	27	11	0	0	0	0	0	0	0	0	0	0	0	0
	A‐T (28; 9.72%)	1	8	4	7	4	2	2	0	0	0	0	0	0	0	0	0	0	0	0
	A‐NT (15; 5.2%)	3	5	1	2	1	2	1	0	0	0	0	0	0	0	0	0	0	0	0
	NA‐T (202; 70.13%)	58	31	26	46	14	20	7	0	0	0	0	0	0	0	0	0	0	0	0
	NA‐NT (43, 14.93%)	20	9	4	3	3	3	1	0	0	0	0	0	0	0	0	0	0	0	0
Progressive disease (132, 31.43%)	PP (30; 22.73%)	0	0	0	0	0	0	0	18	3	9	12	13	20	9	8	3	3	4	0
	SP (102; 77.27%)	0	0	0	0	0	4	4	0	1	0	4	6	5	1	2	2	0	0	1
	A‐NP (8; 6.06%)	0	0	0	0	0	0	1	2	0	2	0	1	1	0	0	0	1	0	0
	A‐P (23; 17.42%)	0	0	0	0	0	1	1	3	1	1	4	2	5	4	1	0	0	0	0
	NA‐P (48; 36.36%)	0	0	0	0	0	1	2	9	2	5	9	8	11	2	2	1	0	1	0
	NA‐NP (53; 40.15%)	0	0	0	0	0	2	0	4	1	1	3	8	8	4	7	4	2	3	1

Note

Each column indicates how many patients with that exact EDSS score are in a given disease type or disease activity subgroup.

Abbreviations: A‐NP, active–not progressive (patient showing disease without progression); A‐NT, active–not treated (patients showing disease activity without receiving treatment); A‐P, active–progressive (patient showing disease and progression at the same time); A‐T, active–treated (patients showing disease activity despite receiving treatment); CIS, clinically isolated syndrome; NA‐NP, not active–not progressive (patient not showing disease activity nor progression); NA‐NT, not active–not treated (patient not showing disease activity without receiving treatment); NA‐P, not active–progressive (patient not showing disease activity while showing progression); NA‐T, not active–treated (patient not showing disease activity while receiving treatment); PP, primary progressive disease; R‐R, relapsing–remitting; SP, secondary progressive disease.

Progressive disease type was identified in 132 of our patients (31.43% of the total population), their average disease duration, age, and age at diagnosis were 20.77 (±12.20), 58.4 (±11.5), and 37.6 (±11.3) years, and mean EDSS score was 5.92 (±1.46) points. Disease activity could be established in 31 patients and 23 persons' (17.42%) disease showed progression adjacent to activity (active–progressive arm, A‐P), while progression was not seen despite disease activity in eight patients (6.06%, active–not progressive arm, A‐NP). Mean age, age at diagnosis, disease duration, and EDSS score were 49.91 (±10.46), 34.74 (±10.35), 15.17 (±10.49) years, and 5.65 (±1.28) points in the A‐P arm and were 49.38 (±8.99), 35.13 (±13.23), 14.25 (±10.08) years, and 5.31 (±1.65) points in the A‐NP arm. No disease activity, nor progression (not active–not progressive arm, NA‐NP) could be identified in 53 (40.15%) patients, while progression could be determined without signs of activity in 48 (36.36%) patients. Average age, age at diagnosis, disease duration, and EDSS score were 59.98 (±10.05), 36.85 (±10.11), 23.00 (±12.04) years, and 5.57 (±1.23) points in the NA‐NP group and 62.33 (±11.16), 40.17 (±12.49), 22.06 (±12.56) years, and 6.54 (±1.56) points in the NA‐P group, respectively (Tables 2 and 3). There was no difference in distribution in‐between genders (*p* = .258) regarding disease phenotypes (data not shown).

A total of 341 patients (81.19% of the total cohort) had received treatment at some point during their lifetime, and on the prevalence day, 312 (74.28%) of our patients were treated. A total of 230 (79.86%) patients with an R‐R disease type, and 72 patients (54.54%) with progressive disease type received treatment on the prevalence day. A total of 178 patients received platform therapies, and 134 were prescribed a HADMT. As a first choice, 307 patients received platform drugs and 34 patients were started on a HADMT from the beginning of their treatment (Table 4).

**Table 4:** Present and past treatment status of our patients by disease type and disease activity

			Treated ever		Treated now			Started on HADMT		Started on platform	
			No	Yes	No	Platform	HADMT	No	Yes	No	Yes
R‐R disease (288, 68.57%)	CIS (12; 4.16%)	NA‐NT	12	0	12	0	0	12	0	12	0
	R‐R (276; 95.84%)	A‐T	0	28	0	10	18	27	1	1	27
		A‐NT	12	3	15	0	0	15	0	12	3
		NA‐T	0	202	0	134	68	182	20	20	182
		NA‐NT	26	5	31	0	0	31	0	26	5
Progressive disease (132, 31.43%)	PP (30; 22.73%)	A‐NP	0	2	0	1	1	1	1	1	1
		A‐P	3	3	3	0	3	4	2	5	1
		NA‐NP	2	5	2	1	4	4	3	5	2
		NA‐P	7	8	8	0	7	9	6	13	2
	SP (102; 77.27%)	A‐NP	0	6	1	2	3	5	1	1	5
		A‐P	1	16	5	3	9	17	0	1	16
		NA‐NP	4	42	7	22	17	46	0	4	42
		NA‐P	12	21	24	5	4	33	0	12	21

Note

Columns one and two show how many patients in each subgroup have ever been treated during some point in their disease, and the potency of the used DMT for patients who are treated on the prevalence day. Columns three and four show how many patients in each subgroup have started their treatment with low or high potency drugs. Most of the still treated R‐R patients started their treatment with platform drugs, and escalation to a HADMT was necessary with 48 of them. On the contrary, a higher fraction of patients with a progressive disease started their treatment with a HADMT. Furthermore, a bigger ratio of PP and SP patients switched onto a HADMT after being started on platform drugs, than did patients in the R‐R disease type group.

Abbreviations: A‐NP, active–not progressive (patient showing disease without progression); A‐NT, active–not treated (patients showing disease activity without receiving treatment); A‐P, active–progressive (patient showing disease and progression at the same time); A‐T, active–treated (patients showing disease activity despite receiving treatment); CIS, clinically isolated syndrome; HADMT, highly active disease modifying treatment—fingolimod, natalizumab, ocrelizumab, cladribine, alemtuzumab, and mitoxantrone; NA‐NP, not active–not progressive (patient not showing disease activity nor progression); NA‐NT, not active–not treated (patient not showing disease activity without receiving treatment); NA‐P, not active–progressive (patient not showing disease activity while showing progression); NA‐T, not active–treated (patient not showing disease activity while receiving treatment); Platform, platform therapies—interferons, glatiramer acetate, dimethyl fumarate, and teriflunomide; PP, primary progressive disease; R‐R, relapsing–remitting; SP, secondary progressive disease.

Of the 28 R‐R patients whose disease showed activity despite treatment, 10 patients were treated with platform drugs, 18 with HADMTs. Four patients have refused to change and escalate treatment despite having an active disease while using a DMT, 2 patients have commenced a DMT <1 month before the prevalence day; hence, their disease was still considered active and 22 patients have been switched to another DMT within 3 months of the prevalence day (i.e., on their next visit). From the 15 patients in the active–not treated arm, three patients began treatment shortly after the prevalence day, one patient has given birth not much prior to the prevalence day and has restarted treatment later, and the rest, 11 patients, either refused treatment or were unable to be treated because of compliance issues. Of the 43 patients that showed no signs of disease activity despite being untreated 20 patients had an isolated attack comprising of optical neuritis and thus were closely observed and given no treatment yet, one patient was started on a DMT shortly after the prevalence day, while five people have been treated with platform drugs throughout their disease, but treatment have been ceased with all of them (two patients became pregnant, one patient developed a malignant disease, one patient asked for the discontinuation of treatment, and one patient was unfit to be treated due to compliance issues). Of the 202 patients in the NA‐NT arm, 134 received platform drugs and 68 HADMTs. Regarding the progressive disease phenotype, 15 people were treated out of the 17 patients with an active–progressive disease; 12 people were prescribed a HADMT, and three were administered platform therapies. Almost all, seven out eight patients were treated in the active–not progressive arm, three patients with platform drugs, and four with HADMTs. In the not active–not progressive and not active–progressive groups, 23 and five patients received platform drugs, 21 and 11 people were administered HADMTs, respectively (Table 4). The majority of our patients (134) were using their first choice of DMTs, 103 patients underwent treatment change once, 50 people twice and 23 patients three times. Four and five treatment changes were necessary with 1–1 patient (Table 5).

**Table 5:** Number of treatment changes during the patients' disease course stratified by disease type and disease activity

			Number of changes					
			0	1	2	3	4	5
R‐R disease (288, 68.57%)	R‐R (276; 95.84%)	A‐T	7	11	8	1	0	1
		A‐NT	1	2	0	0	0	0
		NA‐T	96	66	26	13	1	0
		NA‐NT	3	1	1	0	0	0
Progressive disease (132, 31.43%)	PP (30; 22.73%)	A‐NP	1	0	0	1	0	0
		A‐P	2	0	1	0	0	0
		NA‐NP	4	0	1	0	0	0
		NA‐P	7	1	0	0	0	0
	SP (102; 77.27%)	A‐NP	1	3	2	0	0	0
		A‐P	4	5	5	2	0	0
		NA‐NP	15	15	6	6	0	0
		NA‐P	10	5	5	1	0	0

Note

In the majority of patients with whom no disease activity was seen, it was achieved with either their first or second disease modifying treatment (NA‐T and NA‐NP groups within the R‐R and progressive disease course, respectively).

Abbreviations: A‐NP, active–not progressive (patient showing disease without progression); A‐NT, active–not treated (patients showing disease activity without receiving treatment); A‐P, active–progressive (patient showing disease and progression at the same time); A‐T, active–treated (patients showing disease activity despite receiving treatment); CIS, clinically isolated syndrome; NA‐NP, not active–not progressive (patient not showing disease activity nor progression); NA‐NT, not active–not treated (patient not showing disease activity without receiving treatment); NA‐P, not active–progressive (patient not showing disease activity while showing progression); NA‐T, not active–treated (patient not showing disease activity while receiving treatment); PP, primary progressive disease; R‐R, relapsing–remitting; SP, secondary progressive disease.

## 4 DISCUSSION

In our current study, the male/female ratio was 1:2.925 in the MS population and 1:1.106 in the county population, and the standardized prevalence was 53.9/100,000 for men and 144.8/100,000 for women. The total incidence of MS for the examined period was 4.44/100,000, while it was 2.44/100,000 for men and 6.25/100,000 for women. This makes Hungary a medium‐risk country for MS in accordance with previous epidemiological studies from this region (Benjak et al., 2018; Kapica‐Topczewska et al., 2018; Salhofer‐Polanyi et al., 2017; Zsiros et al., 2014).

Examining the same area, Bencsik et al. measured the male/female ratio to be 1:2.75 and 1:3.08 in the MS population and 1:1.09 and 1:1.12 in the overall county population in 1999 and 2013, respectively (Bencsik et al., 2001; Zsiros et al., 2014). Our results are in accordance with current findings in the literature regarding the continuous rising of prevalence of MS in the past decades, as well as higher occurrence of MS in women (Benito‐Leon, 2011; Kingwell et al., 2013; Koch‐Henriksen, Thygesen, Stenager, Laursen, & Magyari, 2018). Even though there is no obvious explanation yet for this tendency, many factors have been suspected to play a role in it (Koch‐Henriksen et al., 2018). The presumed factors of decreased amount of childbirth (Hungarian Central Statistical Office, 2019), increasing occurrence and severity of obesity (Leray et al., 2010), and high tobacco consumption (Alpar et al., 2016) are valid for Hungary as well. Also, the new McDonald^1^ criteria make the diagnosis of definite MS possible faster than before. In addition, since the last epidemiological study from the same region not only several HADMTs have entered the market but all of them have been reimbursed by the healthcare provider, rendering the whole therapeutic palette accessible for every patient. This made a personalized, tailored to disease activity treatment available for everyone, considerably prolonging the time to conversion to a secondary progressive disease even for patients with very high disease activity, therefore increasing the overall survival of the patients. We presume that all these factors may play a role in the seen increase of both prevalence and incidence of MS in the surveyed region.

The distribution of our patients based on their disease course (65.71% had relapsing–remitting, 24.29% had secondary progressive, 7.14% had primary progressive disease course and 2.86% of our patients were diagnosed with CIS) is also in line with recently published data based on large registries from both high and low prevalence regions for MS from across Europe, from Finland, Italy, Argentina, and Sweden (Hillert & Stawiarz, 2015; Laakso et al., 2019; Mellinger et al., 2018; Pirttisalo, Soilu‐Hanninen, & Sipila, 2019; Trojano et al., 2019; Urru, Antonelli, & Sechi, 2019).

According to the phenotypic classification (Lublin, 2014), roughly 2/3rd of our patients (288 patients, 68.57%) had a relapsing–remitting disease type and 1/3rd were diagnosed with a progressive disease (132 patients, 31.43%). The majority (83.33%) of patients with a relapsing–remitting disease course received treatment. Most of them were successfully treated according to their disease activity as 87.8% of the patients treated showed no disease activity with their current DMT, also their mean EDSS (1.39) was low compared to their relatively long mean disease duration (11.55 years). Most of the patients with a relapsing–remitting disease course were using platform therapies (62.6%), roughly one third of them were treated with a HADMT (37.4%).

On the prevalence day, 62.1% of the patients with a progressive disease were treated, more people received a HADMT (58.5%), than platform therapies (41.5%). Compared to patients with an R‐R disease course, an even bigger ratio of patients (87.4%) started their treatment with platform drugs, as previously no therapy was approved for primary progressive disease, also when most patients, who now have a SP disease began their treatment at a time when only platform drugs were available for the R‐R disease type and converted before the introduction of HADMTs. The currently seen high number of patients on a HADMT among patients with a progressive disease is owed to the recent approval of ocrelizumab for the treatment of primary progressive disease in Hungary. Regarding the whole cohort, an escalative therapeutic approach was used in the history of most patients, as at the time when we began to treat most of our patients HADMTs were either not available yet at all or were not reimbursed by the healthcare provider as a first choice of treatment; therefore, only a lateral change or escalation was possible, induction with a highly potent drug was not.

In the near future, an even higher ratio of patients is expected to be using HADMTs (especially among patients with progressive disease), as more patients are going to be diagnosed with a secondary progressive disease type, than nowadays, not only due to the introduction of the new diagnostic criteria for secondary progressive MS, but because several highly active DMTs are already in the pipeline awaiting EMA approval for the treatment of SPMS.

A limitation of our study is the relatively small sample size, however taking into consideration that only our clinic maintains an MS registry in the country and that the examined area comprises of 4% of the country's total population, and all our diagnostic tools and treatment options are available countrywide, our findings can be considered as representative for the whole of the MS population in Hungary. Strong points include the good quality data extracted from our registry, and our current study appears to be one of the first epidemiological studies that have used the most recent McDonald criteria as well as the newly proposed phenotypic classification of disease type, and one that has also evaluated the treatment status of the patients. Other strong point is that because of a previous epidemiological study in this area (Bencsik et al., 2001; Zsiros et al., 2014) it provides a picture of disease development. Thus, it gives valuable information to the healthcare provider not only about the size of the population that needs to be treated, but of the actual status of their disease and efficacy of the used therapy, and possible future therapeutic and financing needs. Furthermore, fresh epidemiological data from the Central European region based on recent diagnostic and classification criteria were lacking, which demand our study addresses.

## 5 CONCLUSION

The new phenotype–based classification system, new therapeutic guidelines and the most recent revisions to the McDonald diagnostic criteria invoke the need for a fundamentally different therapeutic approach than used before. In contrast to the previous escalative practice, a personalized treatment strategy is urged. To achieve this, the constant re‐evaluation of the patient's disease course along with disease activity is needed. With the timely start of an adequate DMT and rapid changes in treatment, when necessary, long‐term stability and significant slowing of disease progression can be achieved not only in patients with R‐R, but with progressive disease as well.

## CONFLICT OF INTEREST

The authors report no conflict of interest.

## AUTHOR CONTRIBUTIONS

T.B., D.S., and K.B. conceived and designed the study. T.B., D.S., Zs.F.‐N., Zs.T.K., J.F., R.L., and Zs.K. recruited patients and gathered patient data. T.B. analyzed the data and wrote the paper. K.B., L.V., and P.K. edited and revised the manuscript. All authors read and approved the final manuscript.

## Data Availability

The data that support the findings of this study are available from the corresponding author upon reasonable request.
